# G protein–coupled receptor interactions with arrestins and GPCR kinases: The unresolved issue of signal bias

**DOI:** 10.1016/j.jbc.2022.102279

**Published:** 2022-07-19

**Authors:** Qiuyan Chen, John J.G. Tesmer

**Affiliations:** Departments of Biological Sciences and of Medicinal Chemistry and Molecular Pharmacology, Purdue University, West Lafayette, Indiana, USA

**Keywords:** arrestin, G protein-coupled receptor, GPCR kinase, GRK, cryo-electron microscopy, single particle reconstruction, biased agonism, desensitization, allostery, Arr1, arrestin-1, Arr2, arrestin-2, Arr3, arrestin-3, AST, active site tether, GPCR, G protein-coupled receptor, GRK, G protein-coupled receptor kinase, H8, helix 8, ICL, intracellular loop, M_2_R, M2 muscarinic receptor, MD, molecular dynamics, NTSR_1_, neurotensin receptor 1, RH, regulator of G protein signaling-homology, Rho, rhodopsin, TM, transmembrane, V_2_R, vasopressin 2 receptor, V_2_Rpp, vasopressin 2 receptor–derived phosphopeptide, β_1_AR, β1 adrenergic receptor

## Abstract

G protein–coupled receptor (GPCR) kinases (GRKs) and arrestins interact with agonist-bound GPCRs to promote receptor desensitization and downregulation. They also trigger signaling cascades distinct from those of heterotrimeric G proteins. Biased agonists for GPCRs that favor either heterotrimeric G protein or GRK/arrestin signaling are of profound pharmacological interest because they could usher in a new generation of drugs with greatly reduced side effects. One mechanism by which biased agonism might occur is by stabilizing receptor conformations that preferentially bind to GRKs and/or arrestins. In this review, we explore this idea by comparing structures of GPCRs bound to heterotrimeric G proteins with those of the same GPCRs in complex with arrestins and GRKs. The arrestin and GRK complexes all exhibit high conformational heterogeneity, which is likely a consequence of their unusual ability to adapt and bind to hundreds of different GPCRs. This dynamic behavior, along with the experimental tactics required to stabilize GPCR complexes for biophysical analysis, confounds these comparisons, but some possible molecular mechanisms of bias are beginning to emerge. We also examine if and how the recent structures advance our understanding of how arrestins parse the “phosphorylation barcodes” installed in the intracellular loops and tails of GPCRs by GRKs. In the future, structural analyses of arrestins in complex with intact receptors that have well-defined native phosphorylation barcodes, such as those installed by the two nonvisual subfamilies of GRKs, will be particularly illuminating.

It is now widely appreciated that G protein–coupled receptors (GPCRs) instigate intracellular signaling by one of two transducer families, either heterotrimeric G proteins or GPCR kinases (GRKs). Heterotrimeric G proteins are GTP-dependent switches that are activated when GPCRs on the cell surface interact with either natural environmental cues or synthetic agonists used for therapeutic or recreational purposes. In their active, GTP-bound state, heterotrimeric G proteins dissociate into a Gα·GTP subunit and a Gβγ heterodimer that can independently interact with effector enzymes or ion channels to provoke an appropriate cellular response. There are various mechanisms by which cells fine tune GPCR signaling so that they can not only remain responsive to changes in environmental cues but also avoid damage from sustained signaling. The primary way this occurs at the level of the receptor is *via* the process of homologous desensitization, which is initiated by the GRKs (1, 2, 3). Like heterotrimeric G proteins, GRKs selectively interact with active GPCRs. GRKs first phosphorylate clusters of sites in an extended intracellular loop (ICL) or the C-tail of the activated GPCR. Arrestins then bind to these phosphorylated clusters, and sometimes also to the activated transmembrane (TM) core of the receptor, leading to a conformational change in the arrestin. In the vertebrate visual signaling cascade, arrestin binding chiefly serves to block the binding of additional G proteins to rhodopsin (Rho). In hormone responsive GPCRs, arrestin binding also targets these receptors for clathrin-mediated endocytosis and internalization, which decreases the number of active receptors on the cell surface (a process called downregulation). The bound arrestin and/or the process of receptor internalization can also trigger novel signaling pathways (4). Furthermore, some arrestin-bound receptors continue to signal from endosomes *via* the heterotrimeric G proteins and/or effector enzymes that internalize with them (5).

In their native context, activated GPCRs can exhibit bias toward either canonical (G protein) or GRK–arrestin pathways (6) or even toward individual members of these families. Such bias can derive from spatiotemporal factors, modulatory proteins, differential expression levels of transducers across different cell types and tissues, and the intrinsic bias of the receptor itself. These have all been categorized as forms of “system bias”. Another form of bias, termed “ligand bias”, is the ability of some receptor agonists to promote one downstream pathway over another relative to a reference agonist (7, 8, 9). The promise of ligand bias is that it could be exploited to deemphasize or eliminate signaling pathways associated with unwanted side effects, thereby engendering a new generation of safer drugs (10, 11). Ligand bias is thought to be driven by allosteric changes in the receptor itself. For example, studies on the angiotensin II type 1 receptor reveal discrete conformations in the core of the receptor as a function of the bias of the bound agonist (12). But how these ligands reconfigure the cytoplasmic region of the GPCR to selectively favor the binding of G proteins, GRKs, or arrestins is not understood.

In this JBC review, we focus on five recent cryo-EM structures of GPCRs in complex with an arrestin and the first near-atomic structure of a GPCR in complex with a GRK. Keeping in mind the caveats imposed by the technical tricks used to achieve these structures, we investigate whether there might be general structural features in these complexes that contribute to either intrinsic bias or result from ligand bias that favors GRK/arrestin signaling. We also assess how these structures advance our understanding of phosphorylation “barcodes” installed by GRKs and other kinases, which can on their own promote bias by provoking distinct conformations of arrestin or different GPCR-arrestin configurations.

## Arrestins and their receptor and membrane-binding elements

**Figure 1:**
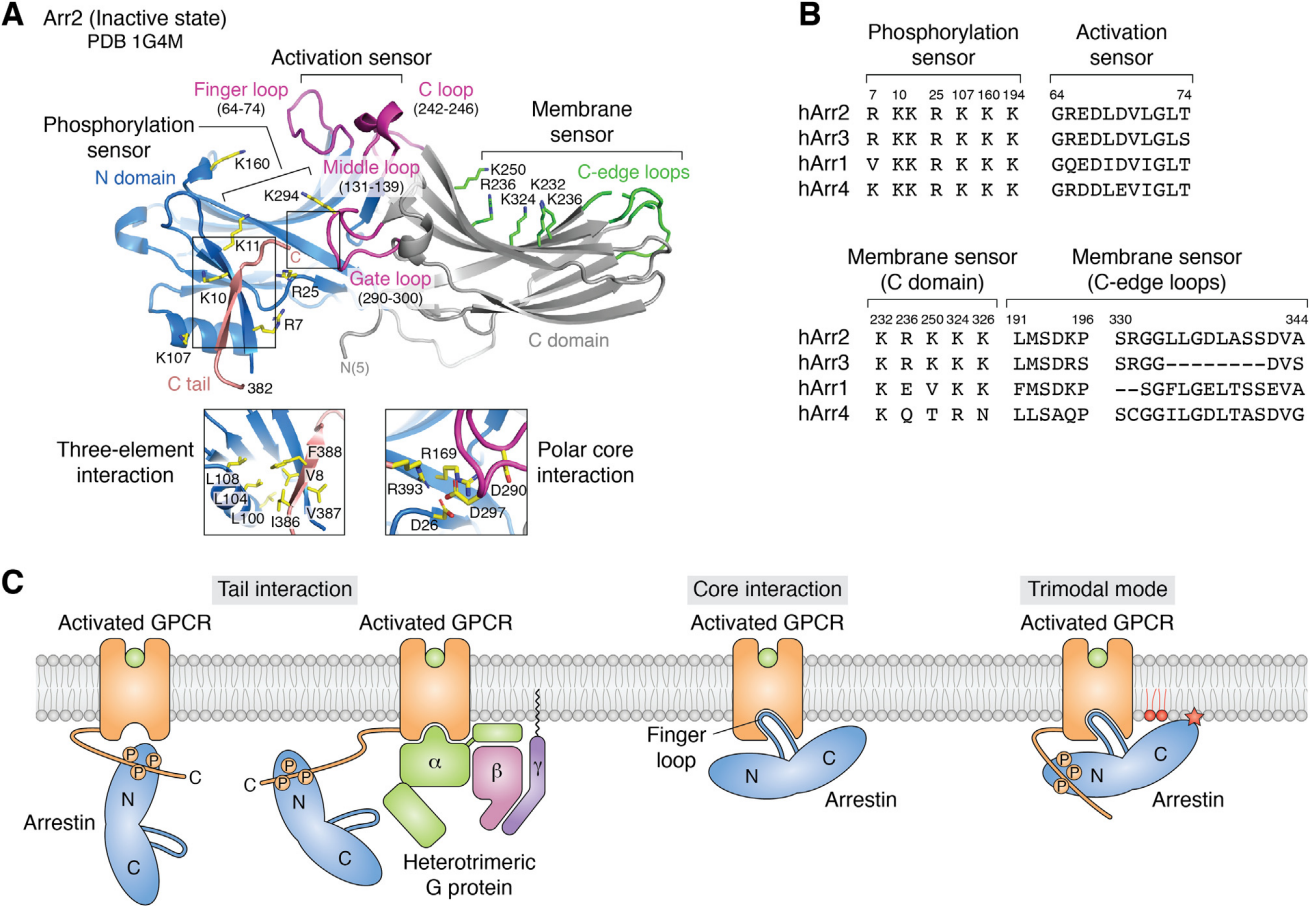
**Arrestins contain conserved structural elements that serve as sensors for active, phosphorylated GPCRs and their surrounding anionic lipid environment.***A*, structure of Arr2 in its basal, inactive state (PDB entry 1G4M) (13). Details of two interdomain interactions stabilizing this state are highlighted in the insets. Named loops mentioned in the review are shown in *purple* with corresponding residue ranges. Structural elements serving as the phosphorylation and membrane sensors are highlighted in *yellow* and *green*, respectively. The activation sensor includes the finger loop and the groove it forms primarily with the C loop. *B*, sequence alignment of human arrestin phosphorylation, activation, and membrane sensors. Residue numbering is based on human Arr2. *C*, cartoon representation of the tail interaction, core interaction, and trimodal mode formed between activated GPCRs, arrestins, and the membrane. Arr2, arrestin-2; GPCR, G protein-coupled receptor.

There are four members of the arrestin family. Arrestin-1 (Arr1) and arrestin-4 are highly expressed in the visual system, whereas arrestin-2 (Arr2; β-arrestin 1) and arrestin-3 (Arr3; β-arrestin 2) are found in all other tissues. Arr2 and Arr3 share >75% sequence identity, and their basal, inactive states are very similar in that they have a r.m.s.d. of 1.2 Å over 393 Cα atoms (13, 14). They contain two domains (N and C) that undergo a ∼20˚ rotation with respect to each other upon full activation (15). Their basal state is stabilized primarily by two inter-domain interactions known as the “polar core” and the “three-element interaction” ((16), in particular see Fig. 3*C* therein) (Fig. 1*A*). The C tail of arrestin binds to the N domain and contributes to both interactions, thus favoring the basal state, but is released upon activation such that it can then interact with proteins mediating endocytosis such as AP2 and clathrin.

Arrestins contain two to three structural elements that function as sensors for phosphorylated, active GPCRs that are in an appropriate lipid environment (Fig. 1*A*). The “phosphorylation sensor” is a cluster of lysines and arginines on the N domain that are shielded by the C tail in the basal state. These residues interact with phosphorylated clusters of Ser and Thr residues in the GPCR, which are typically found in the C tail but sometimes in ICL3 when it is particularly extended. This interaction was first structurally described in crystal structures of Arr2 with a vasopressin 2 receptor (V_2_R)–derived phosphopeptide (V_2_Rpp) (17) and of Arr1 in complex with phosphorylated active Rho (18). The “activation sensor” is defined by the finger loop and the shallow groove it forms with the adjacent C loop (19). The finger loop is flexible in the basal state and can insert into the cytoplasmic cleft formed in activated GPCRs when a core interaction is formed. The term “activation” here does not refer to the activation state of arrestin but instead the fact that the sensor can recognize the active configuration of the TM core. The “membrane sensor” is comprised of a cluster of lysines and arginines on the C domain and a mixture of hydrophobic and hydrophilic residues on the two “C-edge” loops. These residues bind to phosphoinositide head groups or directly insert into the hydrophobic layer of cell membrane, respectively, thereby promoting tighter receptor binding. The phosphoinositide-binding sites were first identified biochemically (20) and are predicted to overlap with those of inositol hexakisphosphate in Arr2/3·IP_6_ complexes (21, 22). The ability of “C-edge” loops to serve as membrane anchors was first suggested by the structure of the Rho–Arr1 complex (19) and further validated using molecular dynamics (MD) simulation and fluorescence quenching (23). The phosphorylation and activation sensors are well conserved among arrestin isoforms, whereas the membrane sensor is variable (Fig. 1*B*).

Although the phosphorylation and activation sensors make direct contacts with receptors and therefore contribute to the affinity and selectivity of the complex, their contributions to arrestin activation likely vary from receptor to receptor. Functional and computational studies indicate that engagement of either or both these sensors can promote arrestin activation (24). Within the phosphorylation sensor, biochemical and structural studies point toward a pair of basic residues in β-strand I of the N domain (*e.g.*, Lys10 and 11 in Arr2) as the most important for phosphate-mediated activation of arrestin (25, 26) perhaps because they form not only hydrophobic contacts in the three-element interaction with the arrestin C-tail but also electrostatic interactions with multiple phosphorylated residues in the active receptor-bound state. In the activation sensor, the interactions of the receptor with the groove between the finger and C loops of arrestin seems to be one of the key driving forces for receptor core-mediated activation of arrestin (24). It is not yet clear if or how the membrane sensor potentiates arrestin activation.

Given that just two arrestins (Arr2 and Arr3) must interact with hundreds of different GPCRs, a high degree of adaptability is required because they have to find a way to favorably interact with the unique residues and lipid environments presented by each activated receptor, as well as the distinct phosphorylation patterns installed by different GRKs or second messenger kinases (*e.g.*, PKA or PKC). Indeed, the available structural data now show that arrestins are amazingly versatile and can engage receptors in dramatically different ways (27) (Fig. 1*C*). The most conserved interaction in all the available structures is that of the phosphorylation sensor, which engages the phosphorylated residues of the activated receptor in what is termed the “tail interaction” (although in some cases, this instead involves the extended ICL3 of the receptor). When only the tail interaction occurs, a GPCR–arrestin complex can sample many conformations (28) including those that would allow the simultaneous engagement of heterotrimeric G proteins with the core of the receptor (29). The most versatile interaction is made by the activation sensor, which in each of the available structures seems to find a unique way to interact with the activated TM core in what is termed the “core interaction”. It is this interaction, when present, that seems to fix the relative rotation of arrestin relative to the TM helices of the receptor. The membrane sensor binds to the lipids surrounding the GPCR and provides an additional stabilizing anchor to form the “trimodal mode”. This interaction likely contributes to the variable tilt of the bound arrestin with respect to the membrane surface that has been observed among structures. However, note that arrestins do not need to form the full trimodal mode to elicit downstream signaling in cells (30, 31).

## Recent structures of Arr2 bound to hormone-responsive GPCRs

There are now over 286 structures of GPCRs in complex with G proteins (32), but only five of a hormone-activated GPCR in complex with arrestin (Table 1): two with neurotensin receptor 1 (NTSR_1_) (33, 34), one with the M_2_ muscarinic receptor (M_2_R) (35), one with the β_1_ adrenergic receptor (β_1_AR) (36), and the most recent with the V_2_R (37). All these complexes involve Arr2 in a “trimodal mode” interaction, most likely because this arrangement has the highest affinity and exhibits the least dynamics, which typically portends higher resolution data in cryo-EM experiments.

**Table 1:** Summary of GPCR–arrestin/GRK structure determinations

PDB	Res(Å)	Receptor	Ligand	Transducer	GPCR phosphorylation	Fab	Model membrane	Additional modification
4ZWJ	3.3	Opsin	none	Arr1	endogenous kinases in HEK cells	NA^a^	monopalmitolein	T4 Lysozyme-Rho-Arr1 fusion
5W0P	3.0	N2C/N282C E113Q/M257Y		L374A, V375A, F376A			cholesterol	
6UP7	4.2	NTSR_1_	NTS_8–13_	Arr2_1-382_	GRK5, *in vitro*	NA	LMNG/GDN/CHS^b^	Sulfo-NHS-LC-diazirine crosslink
6U1N	4.0	M_2_R	iperoxo/LY2119620	Arr2_1-393_ with minimal Cys^c^	V_2_Rpp^d^ sortase ligation	Fab30	nanodisc (MSP1D1E3)	
6TKO	3.3	β_1_AR (β83)^e^	formoterol	Arr2 L68C, R169E	V_2_R_6P_ sortase ligation^f^	Fab30	nanodisc (Zebrafish apo-lipoprotein A-1)	
6PWC	4.9	NTSR_1_ 49-418	NTS, ML314	Arr2_1-393_ I386A, V387A, F388A V81C, A27C	GRK5 coexpression	Fab30	digitonin	BRIL-NSTR1-Arr2-Fab30 fusion disulfide crosslinking^g^
7R0C	4.7	V_2_R N22E	AVP	Arr2_1-382_	endogenous kinases in Sf9 insect cells	ScFv30	LMNG/GDN/CHS	
7MTA	4.1	Rho	all-trans retinal	GRK1_1-535_ S5E, S488E,T489E	none	Fab1	LMNG	MC4/DC4 crosslink
7MTB	4.0	Rho	all-trans retinal	GRK1_1-535_ S5E, S488E,T489E	none	Fab6	LMNG	MC4/DC4 crosslink

a NA, not applicable.

b LMNG, lauryl maltose neopentyl glycol; CHS, cholesteryl hemisuccinate; GDN, glyco-diosgenin.

c Minimal Cys construct contains seven mutations: C59A, C125S, C140I, C150V, C242V, C251V and C269S.

d V_2_Rpp: GGGARGR[pT]PP[pS]LGPQDE[pS]C[pT][pT]A[pS][pS][pS]LAKDTSS. The first three glycines are added to enable sortase ligation. The rest of V_2_Rpp is the same as the phosphopeptide used in the Arr2–V_2_Rpp–Fab30 crystal structure (PDB ID 4JQI) (18).

e β83 variant contains three truncations: Δ1-32, Δ244-271, and Δ358-483 and nine mutations: M44, M90V, V103C, C116L, E130W, D322K, F327A, F338M, and C358A.

f V_2_R6P: GGGDE[pS]A[pT][pT]A[pS][pS][pS]LAKDTSS. The first three glycines are added to enable sortase ligation. The rest of V_2_Rpp is the same as the phosphopeptide used in the Arr2–V_2_Rpp–Fab30 crystal structure (PDB ID 4JQI) (18).

g Cys mutations were introduced to stabilize the complex: V81C in Arr2 with C277 in ICL3 of NTSR1, A279C in Arr2 with G59C in the heavy chain of Fab30.

**Figure 2:**
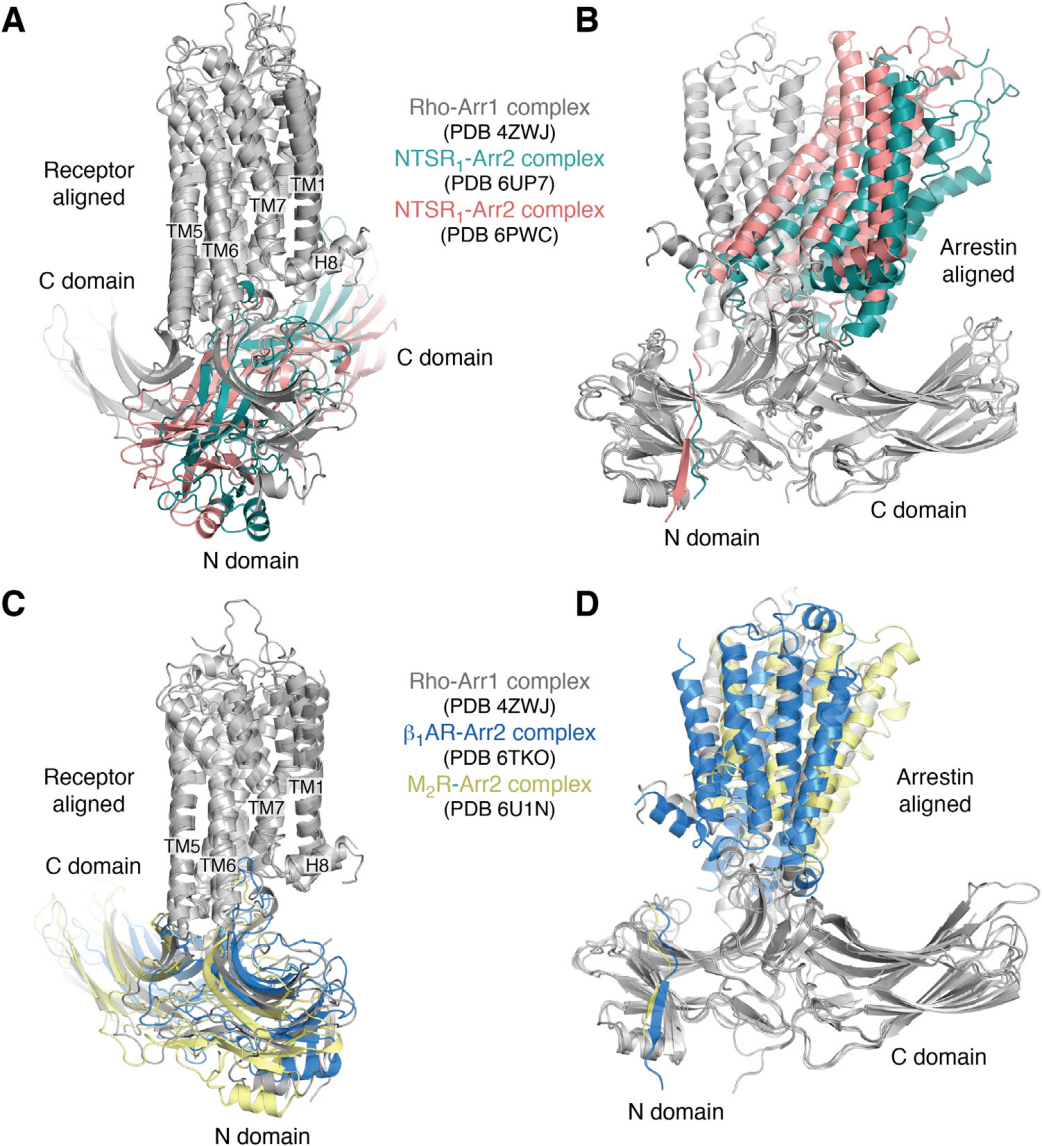
**Conformational heterogeneity in GPCR–arrestin interactions.***A* and *B*, superposition of the Rho–Arr1 (PDB entry 4ZWJ) (19) and NSTR_1_–Arr2 structures (PDB entry 6PWC and 6UP7) (33, 34) with either (*A*) receptor or (*B*) Arr2 aligned. *C* and *D*, superposition of the Rho–Arr1 (PDB entry 4ZWJ) (19), β_1_AR–Arr2 (PDB entry 6TKO) (36), and M_2_R–Arr2 structures (PDB entry 6U1N) (35) with either (*C*) receptor or (*D*) Arr2 aligned. β_1_AR, β1 adrenergic receptor; Arr1, arrestin-1; Arr2, arrestin-2; GPCR, G protein-coupled receptor; H8, helix 8; M_2_R, M2 muscarinic receptor; NTSR_1_, neurotensin receptor 1; Rho, rhodopsin; TM, transmembrane.

The specific differences exhibited by each of the three arrestin receptor/membrane sensors in this series of structures could, in principle, have profound effects on events downstream of arrestin binding, as suggested by MD simulations and fluorescence studies (38). However, the five structural campaigns used approaches that could bias the conformational space of the particles used for 3D reconstructions, potentially obscuring functionally relevant molecular distinctions (Table 1). For example, four out of the five cryo-EM structures were determined in the presence of Fab30 (33, 35, 36) or its single chain variant (ScFv30) (37). Fab30 was originally selected based on its ability to bind activated Arr2 in complex with V_2_Rpp and was used to aid the crystal structure determination of the Arr2–V_2_Rpp complex (17). Four structures utilized preactivated forms of Arr2 created by either truncating its C tail (34, 37), destabilizing the three-element interaction (33), or by disrupting the polar core (36). Two structures used sortase, a protein ligase, to attach a homogenously phosphorylated peptide derived from the V_2_R C tail in place of the native receptor tail (35, 36). Crosslinking (33, 34) or protein fusions (33) were used in two cases. Both NTSR_1_–Arr2 complexes and the V_2_R–Arr2 complex were reconstituted into detergent micelles (33, 34, 37), whereas the M_2_R–Arr2 and β_1_AR–Arr2 complexes were resolved in the more native bilayer-like environment provided by nanodiscs (35, 36). Finally, it should be noted that the established GRK-phosphorylated residues in the M_2_R are in its extended ICL3 (39), and this cannot be replicated by a C-terminal V_2_Rpp fusion protein. The variability that can occur when one uses different technical approaches to determine an EM structure is demonstrated by comparison of the two unique NTSR_1_–Arr2 complexes (33, 34), which exhibit a difference in rotation of 11˚ for Arr2 relative to the receptor (Fig. 2, *A* and *B*). Differences in sample engineering, kinase phosphorylation, complex preparation, and/or decisions made during cryo-EM data processing thus seem to have led to the resolution of two distinct states. Such a high degree of conformational plasticity in the same GPCR–Arr2 complex suggests that it will be difficult to understand arrestin bias by way of cryo-EM if the molecular basis for bias involves only subtle structural differences.

The five recent Arr2 complexes all involve “class A” GPCRs and thus one might have assumed that each structure would end up telling a story similar to that of the Rho–Arr1 complex (19). This is essentially true for the M_2_R–Arr2 and β_1_AR–Arr2 complexes, although they still deviate by about 10° in the relative orientation of arrestin to the receptor (Fig. 2, *C* and *D*). The two NTSR_1_ complexes however reveal a strikingly different orientation of bound Arr2 from the others (Fig. 2, *A* and *B*), with ICL1 of NTSR_1_ binding in place of ICL2 in the activation sensor groove, resulting in a ∼90° rotation of arrestin in the plane of the membrane around the central TM core of the GPCR. Huang *et al.* (34) provide biochemical evidence that NTSR_1_ forms a complex with Arr2 even in the absence of receptor phosphorylation, suggesting that an unusually strong core interaction is formed in this pairing. Yin *et al.* suggest that this new arrestin configuration could be a preferred interaction mode for receptors with phosphorylated ICL3 and short C tails. To investigate this possibility, they studied arrestin complex formation with two such receptors, the HTR1A and B serotonin receptors, *via* disulfide mapping (33). However, inspection of the M_2_R−Arr2 and Rho−Arr1 structures suggest that either Rho-like or NTSR1-like configurations of Arr2 are compatible with the bulk of the crosslinking data (*e.g.*, the crosslink of ICL3 with Arr2-V81C). Furthermore, ICL1 in the serotonin receptors, a structural element that provides a key interaction in the NTSR_1_−Arr2 complexes, is three residues shorter. The most recently reported Arr2 complex, that with the V_2_R, yielded yet another surprising configuration wherein ICL2 is bound in the activation sensor groove, but the body of arrestin is rotated ∼38° around the TM core of the receptor from its position in the Rho, β_1_AR, and M_2_R complexes and ∼54° from that in the NTSR_1_ complexes. Thus, the specific interactions formed by ICL1 and ICL2 allow arrestin to rotate over a wide range of angles relative to the central axis of the receptor TM core, with the finger loop effectively serving as a pivot, at least when the complex is in a trimodal mode.

## The arrestin phosphorylation sensor

**Figure 3:**
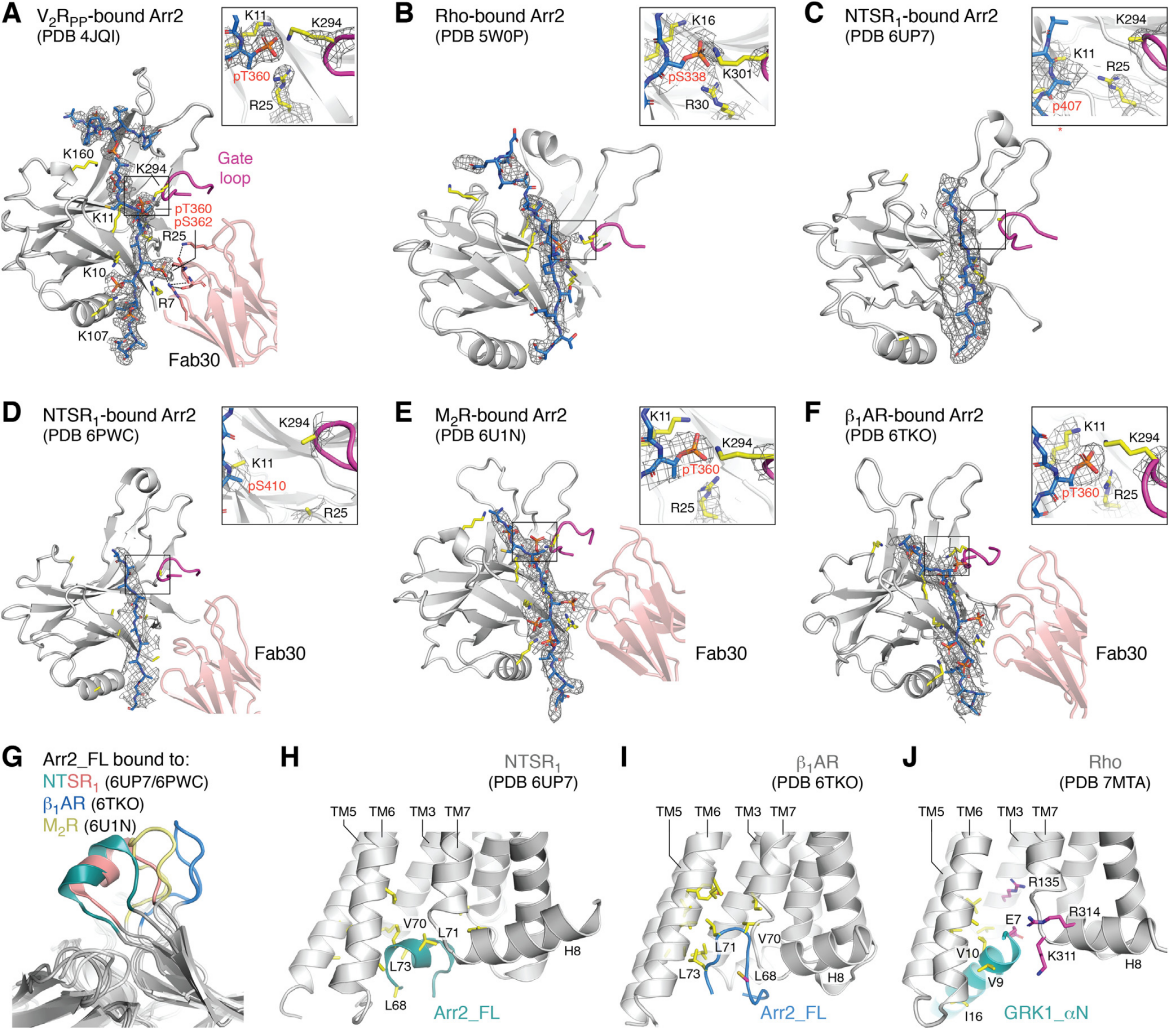
**Comparison of receptor-engaged phosphorylation and activation sensors.***A*–*F*, structures of arrestins bound to (presumably) phosphorylated C tails of GPCRs. When added, Fab30 interacts with the position analogous to pS362 in the V_2_Rpp peptide, effectively stapling arrestin to the GPCR C tail (*D*–*F*). Density for the GPCR C tail in each structure (*gray* wire cages) in general has poor definition, as evidenced by poor stereochemistry in some cases (*e.g.*, see colliding adjacent phosphates in panel *E*). The insets detail the interactions at the most consistently observed phosphosite (corresponding to residue Thr360 in V_2_Rpp in panel *A*), which is coordinated by Arr2-Lys11, Arr2-Arg25, and presumably Arr2-Lys294 (because density is lacking). *G*, the Arr2 finger loop (76) shows many different conformations when bound to a GPCR, highlighting its ability to adapt to distinct cytoplasmic clefts and arrestin orientations relative to the receptor core. Shown is a superposition of Arr2 bound to β_1_AR (PDB entry 6TKO) (36), M_2_R (PDB entry 6U1N) (35), and NTSR_1_ (PDB entry 6PWC and 6UP7) (33, 34). *H* and *I*, interactions of Arr2 finger loop within the cytoplasmic cleft of (*H*) NSTR_1_ (PDB entry 6UP7) (34) and (*I*) β_1_AR (PDB entry 6TKO) (36). *J*, interaction of the GRK1 αN helix with the cytoplasmic cleft of Rho (PDB entry 7MTA) (52). In (*H*–*J*), the side chains of residues contributing hydrophobic and hydrophilic interactions are shown with *yellow* and *magenta* carbons, respectively. β_1_AR, β1 adrenergic receptor; Arr2, arrestin-2; GPCR, G protein-coupled receptor; GRK, G protein-coupled receptor kinase; H8, helix 8; M_2_R, M2 muscarinic receptor; NTSR_1_, neurotensin receptor 1; V_2_Rpp, vasopressin 2 receptor–derived phosphopeptide; Rho, rhodopsin; TM, transmembrane.

Among the recent GPCR–Arr2 complexes, the phosphorylation sensor is similarly engaged by the phosphorylated receptor C tail, forming the classic tail interaction with the Arr2 N domain (Fig. 3, *A*–*F*). Although there is strong density for the backbone atoms in the NTSR_1_ C tail of the two unique structures, neither of the arrestin complexes have sufficient resolution to define the register or phosphorylation status of the bound sequence (Fig. 3, *C* and *D*). The M_2_R and β_1_AR structures used fused phosphopeptides derived from the V_2_R in place of their native C tails, and thus it was assumed that the interactions with the phosphate sensor are the same as in the Arr2–V_2_Rpp–Fab30 crystal structure (17) (Fig. 3, *A*, *E*, and *F*). In the newest V_2_R–Arr2 complex, the receptor tail was purified in a phosphorylated state, but the pattern mapped by mass spectrometry was not agonist dependent nor was the density definitive at all positions in the reconstruction, although it was assumed once again to be similar to the Arr2–V_2_Rpp–Fab30 crystal structure (37). In all but one of the NTSR_1_–Arr2 complexes, the interaction was further stabilized by Fab30, which directly engages with arrestin and the residue corresponding to the pSer362 position in the V_2_Rpp–Fab30 crystal structure (Fig. 3, *A*, *D*–*F*). Among the arrestin complexes, the position corresponding to pThr360 in V_2_R is the most consistently interpreted as a phosphorylated Ser/Thr residue (Fig. 3, *A*–*F*). Here, the phosphate is potentially coordinated by the side chains of Arr2-Lys11, Arr2-Arg25, and Arr2-Lys294. Lys294 resides in the “gate loop” of arrestin, and although the backbone trace of the gate loop is clear, the side chain of Lys294 is not resolved in any of the structures, consistent with the fact that this residue was recently reported to not be important for receptor binding (26). Strong density is more routinely observed among these structures for the side chains of Lys11 and Arg25. Importantly, pThr360 has recently been shown to play critical roles in arrestin recruitment to the V_2_R, where mutation of this residue resulted in G protein bias (40). In a recent crystal structure of Arr3 with a phosphopeptide derived from the C-terminus of CXCR7, this site is unoccupied and in response, Arr3 seems to adopt a partially active conformation (41). Thus, engagement of the site analogous to that which binds pThr360 in the V_2_R may be critical to achieve full arrestin activation by phosphorylated receptors.

The poor density observed for the C tails, particularly for the native termini of the NTSR_1_ complexes, could reflect heterogeneity in binding, phosphorylation, or simply higher dynamics relative to the rest of arrestin. It has previously reported that mutation of specific sets of phosphorylated residues does not eliminate arrestin binding to receptors in cells, as long as additional phosphosites are available (18, 42). Thus, perhaps any phosphorylated peptide can stabilize activated Arr2 at least to some extent. One or more additional phosphosites closely positioned to a V_2_R-pThr360 equivalent phosphosite would be expected to help boost affinity by engaging other basic residues in the phosphorylation sensor. If so, then the influence of specific “phosphorylation barcodes” on the structure of bound arrestins and, ultimately, the nature of their downstream signaling remains an open question (38, 43). Even in the case of the Rho–Arr1 crystal structure, the receptor used was phosphorylated by unknown kinases during its expression in HEK293 cells (18) and the phosphosites retained in its C-tail may not be those important for signaling *in vivo* (44).

## The arrestin activation sensor

The conformation of the finger loop in the activation sensor is heterogeneous among the arrestin complexes (Fig. 3*G*), whereas the C- and middle-loops of the sensor are more consistent. For example, the loop is modeled as a two turn α-helix in the NTSR1 complexes (although it should be noted that the density does not support this interpretation in the Yin *et al.* structure (33)) and instead as an extended loop in the M_2_R and β_1_AR complexes (Fig. 3*G*). Still, in each case, hydrophobic interactions seem to be maintained between the side chains of Arr2-Leu68, Arr2-Val70, Arr2-Leu71, and Arr2-Leu73 with the hydrophobic walls of the cytoplasmic cleft of the receptor, provided primarily by TM helices 5/6 (TM5/6) and ICL2, although details differ (Fig. 3,*H* and *I*). This heterogeneity could be a consequence of the fulcrum-like role of the finger loop as it adapts to how the receptor TM core rotates and tilts to optimize its interactions with the rest of arrestin. Notably, even though the M_2_R and β_1_AR complexes have similar arrestin orientations, their finger loops are structurally distinct from each other (Fig. 3*G*). This could either reflect the fact that their ICL2 loops are different in sequence or that there are distinct lipid interactions formed by the C-edge loops in each preparation. The β_1_AR finger loop could also be influenced by the Arr2-L68C mutation used in this structure determination (Fig. 3*I*).

## The arrestin membrane sensor

All five of the recent structures have the C-edge loops of the Arr2 membrane sensor in contact with or inserted into a nanodisc lipid bilayer or the micelle banding the receptor (33, 34, 35, 36, 37). In the NSTR_1_ structure by Huang *et al.*, basic residues in the Arr2 C domain bind the head group of an ordered phosphatidylinositol-4,5-bisphosphate (34). A similar lipid interaction was also proposed for the V_2_R–Arr2 structure but the density is not as convincing (37). Detergent micelles with a highly curved surface could consequently cause more tilting of Arr2 relative to the GPCR *versus* planar bilayers. Huang *et al.* make the case that flexibility in the orientation of Arr2 relative to the GPCR and/or membrane may be important for it to remain engaged during changes in membrane curvature, such as during endocytosis, or help Arr2 remain in an active configuration at the membrane in the absence of a bound receptor (45) (although it seems more likely that sequestration of the arrestin C-tail during endocytosis might be more relevant for keeping arrestin active). Indeed, both phosphatidylinositol binding and C-edge domain engagement have been shown to be important for the internalization of activated GPCRs such as the β_2_ adrenergic receptor (20), M_2_R (35), and protease-activated receptor 2 (41, 46). Staus *et al.*(35) further report that engagement of the Arr2 membrane sensor potentiates ligand binding and promotes the core interaction with the M_2_R. Because the membrane sensor is not conserved among arrestins, the various arrestin isoforms will necessarily engage the membrane differently, and the strength of the membrane interaction will also likely also depend on the primary sequence, phosphorylation status, and lipid environment of the receptors (47). In fact, Arr3 may not be able to form a trimodal mode.

## GRKs and the structure of the GRK1–Rho complex

**Figure 4:**
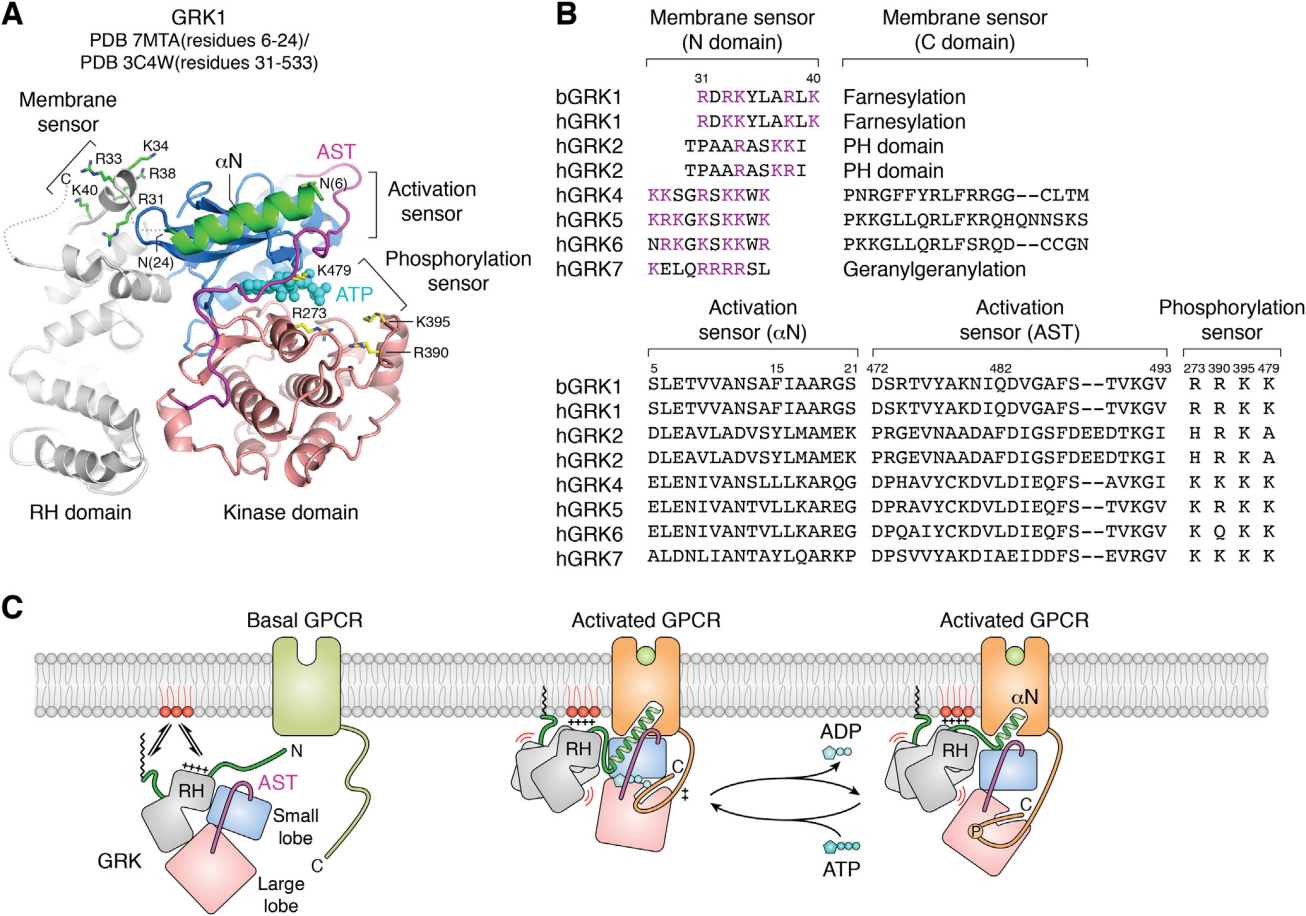
**GRKs also contain conserved structural elements that serve as sensors for active, phosphorylated GPCRs and their surrounding anionic lipid environment.***A*, activated structure of GRK1. The model was generated by merging the GRK1 αN and kinase domain from the structure of GRK1 in complex with Rho (PDB entry 7MTA) (52) and the RH domain from the crystal structure of GRK1 in its basal state (PDB entry 3C4W) (77). The RH domain was in fact disordered in the Rho–GRK1 complex. ATP is modeled in place of the adenosine analog sangivamycin used in the Rho complex. The GRK “membrane” and “phosphorylation” sensors, by loose analogy to those of arrestin, are highlighted with *yellow* and *green* side chains, respectively. The activation sensor is composed of the N-terminal half of αN and adjacent segments of the AST loop (*purple*). *B*, sequence alignment of the GRK phosphorylation, activation, and membrane sensors. Residue numbering is based on bovine GRK1. The N domain membrane sensor mainly interacts with negative phospholipids *via* electrostatic interactions and participating residues are shown in *purple*. Note that a significant role in membrane binding for the residues in this region has not been experimentally demonstrated in GRK2 and 3. *C*, cartoon representation of GRK activation, membrane, and phosphorylation sensors in basal (*left*) and activated, GPCR-bound (*center* and *right*) states. The GRK is speculated to partially dissociate from the receptor during the exchange of ATP, remaining tethered to the receptor either *via* its activation or phosphorylation sensors. AST, active site tether; GPCR, G protein-coupled receptor; GRK, G protein-coupled receptor kinase; Rho, rhodopsin; RH, regulator of G protein signaling-homology.

Bias toward GRK–arrestin pathways will also occur if the receptor adopts a conformation that favors GRK binding over that of heterotrimeric G proteins. Addition of clusters of phosphorylated residues would then promote arrestin binding and inhibit G protein signaling. The catalytic core of all seven mammalian GRKs (GRK1-7) consists of a protein kinase domain inserted into a loop of a regulator of G protein signaling-homology (RH) domain (48) (Fig. 4*A*). An activating PIP_2_-binding site has been identified near the N-terminus of the RH domain in all GRKs except GRK2 and 3, which bind PIP_2_ primarily *via* their pleckstrin homology domains (49, 50, 51, 52). As members of the AGC kinase family, GRKs have many of the structural and regulatory features common to this subfamily, including an extended C-tail that features the “active site tether” (AST), a loop that passes over and contributes residues to the active site (53). However, a distinct element found in all GRKs is an N-terminal helical domain that is typically only ordered in structures of activated GRKs (52, 54, 55), wherein it forms a single α helix (αN) that packs near the hinge of the kinase domain and stabilizes a closed, presumably more active kinase domain conformation. Indeed, truncation (56, 57), antibody blockade (58), or mutation (52, 59) of the N-terminal region dramatically impairs the ability of GRKs to phosphorylate GPCRs. Another difference from most other AGC kinases is the fact that GRKs are not phosphorylated in their activation loops. One evolutionary explanation could be that GRKs have to avoid electrostatic repulsion with partially phosphorylated GPCR polypeptides so that they can install phosphates in clusters, as required by arrestins. Their activity against GPCRs is still however enhanced by autophosphorylation in their AST loop (52). The AST loop only becomes well ordered when the kinase domain adopts more active configurations, wherein it forms part of the binding site for αN (52). AST loop mutations that perturb its observed interactions with αN cause substantial reduction in GRK kinase activity (56). The GRKs are most divergent in their C-terminal regions, which in each case plays a role in membrane localization. GRK1 and 7 (Rho and cone kinase) are farnesylated and geranylgeranylated, respectively, GRK2 and GRK3 bind to Gβγ subunits *via* their pleckstrin homology domain, and GRK4-6 have basic amphipathic helices and/or palmitoylation sites (1) (Fig. 4*B*).

**Figure 5:**
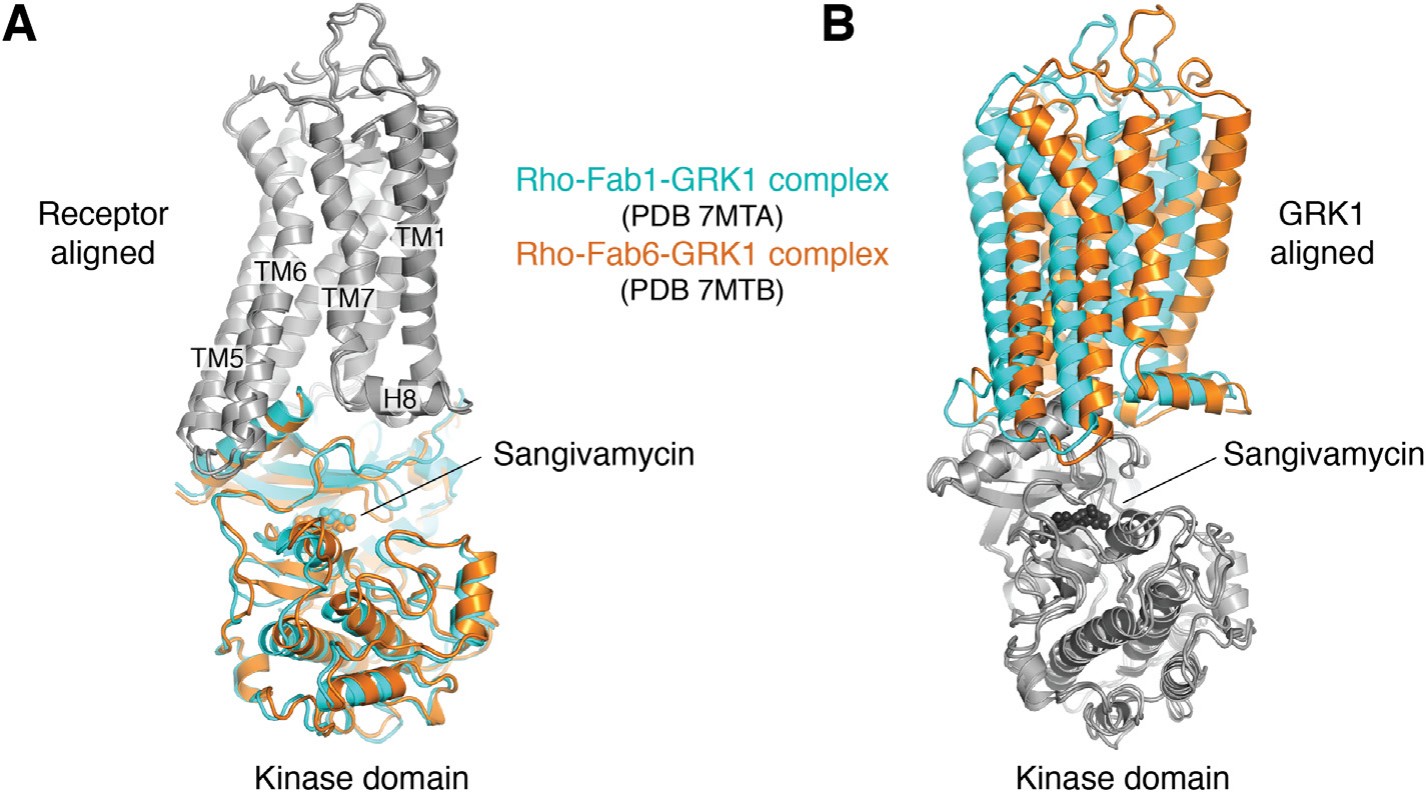
**Conformational heterogeneity in GPCR–GRK1 interactions.** Superposition of the Rho–GRK1 structure in the presence of Fab1 (PDB entry 7MTA) or Fab6 (PDB entry 7MTB) (52) with either (*A*) receptor or (*B*) GRK1 aligned. GPCR, G protein-coupled receptor; GRK, G protein-coupled receptor kinase; H8, helix 8; Rho, rhodopsin; TM, transmembrane.

Recent 4 Å cryo-EM structures of GRK1 in complex with light-activated Rho demonstrate that the N-terminal end of the αN helix directly inserts within the cytoplasmic cleft of the activated receptor (Figs. 3J, 4C and 5). The receptor in turn stabilizes the active conformation of the bound kinase domain by supporting the interactions of the αN helix with the kinase domain hinge (52). A high degree of dynamics and/or conformational heterogeneity was evidenced by the fact that structures derived from two different Fab complexes yielded two distinct configurations of the complex (Fig. 5). The interaction footprint of GRK1 with Rho is distinct from that of arrestins or G proteins in that it forms more extensive interactions with ICL1, which is highly basic in most class A GPCRs (32). These basic residues are predicted to be engaged by the conserved autophosphorylation sites or acidic residues in the AST loop of the kinase domain (52). It remains to be seen if different GRKs in complex with the same receptor or the same GRK in complex with different receptors will exhibit the same range of configurations as does Arr2, but instead using the αN helix as the fulcrum. This seems likely to be the case considering the structural diversity of the broader seven TM receptor family, especially class C and F receptors (60, 61, 62, 63, 64, 65) which have distinctive cytoplasmic clefts but still can be selectively phosphorylated by GRKs.

Although the GRK RH domain can mildly autoinhibit kinase activity by constraining the orientation of the small and large lobes in GRK5 and GRK6 (49, 59, 66) and bind activated Gα_q_ subunits in GRK2/3 (67), its functional role with regards to GPCR phosphorylation remains poorly defined (68). Interestingly, the RH domain is disordered in the Rho–GRK1 complex, perhaps because of otherwise incompatible interactions with ICL2 (52) (Fig. 4*C*). Dynamic behavior was predicted for the RH domain of GRK5 in receptor complexes based on MD, results from cross linking with mass spectrometry, and the small boost in activity that comes from interrupting contacts between the RH domain and the kinase large lobe (49, 66). However, it remains possible that the GRK1 RH domain is already highly dynamic in solution (*i.e.*, in a noncrystalline state) or if its dynamics are simply dependent on the ligand bound in the kinase active site.

## The GRK1 phosphorylation sensor

One can reimagine GRKs as having phosphorylation, activation, and membrane sensors analogous to those of arrestins (Fig. 4, *A* and *B*). Although GRKs are responsible for installing phosphates into active GPCRs, they typically do this in regions where there are dense clusters of Ser/Thr residues. Thus, GRKs must be able to efficiently interact with substrate polypeptides in several different phosphorylation states. The region responsible is a basic patch of residues that line the polypeptide-binding region of the kinase domain (*e.g.*, residues Arg273, Arg390, Lys395, and Lys479 of GRK1), whose side chains are analogous but generally opposite in charge to residues that interact with basic residues in the consensus sequence of PKA substrates (Phe129, Glu203, Asp241, and Asp328, respectively). Consistent with this hypothesis, GRKs tend to favor the phosphorylation of acidic peptides (69), and cross-linking with mass spectrometry data demonstrates proximity between the receptor tail and the various lysine residues that line this region (52). It is not established whether GRKs are processive, but there is some evidence to suggest that GRKs must at least partially dissociate from the receptor core to reload with ATP. It is possible that the receptor ICL3 or C tail may remain bound to the GRK in this semi-dissociated state (52) (Fig. 4*C*). Thus, GRKs could potentially also have tail and core interactions with receptors analogous to those of arrestins (Fig. 1*C*).

## The GRK activation sensor

The N terminal ∼20 amino acids and AST loop of GRK1 are analogous to the activation sensor of arrestin (Fig. 4*A*). Like the arrestin finger loop, these regions are flexible in most inactive GRK structures. In the Rho complex, the αN helix inserts into the cytoplasmic cleft in a manner highly reminiscent of the C terminus of Gα, although with the opposite polarity (52) (Fig. 3*J*). αN makes hydrophobic interactions with the extended TM5 and TM6 helices and contacts the C-terminal end of TM7 and N-terminal end of helix 8 (H8). On the C-terminal end of the αN helix, its residues engage a groove formed between the small lobe, the AST loop, and the large lobe of the GRK kinase domain, which in turn stabilizes the active conformation of the kinase domain. The N-terminus is highly conserved among the GRK family (Fig. 4*B*), suggesting all GRKs use a similar mechanism to both interrogate whether a receptor is active and to become activated upon binding. In support of this idea, the N-termini of GRK5 and GRK6 have also been observed to fold into a helix that forms analogous interactions with its kinase domain (54, 55). Importantly, this was observed when GRK5 is bound to the nonreceptor activator Ca^2+^·CaM (55). Thus, both receptor and nonreceptor activators of GRKs characterized so far use the same general mechanism of kinase activation. Meanwhile, the AST region of GRK1 forms also contacts with ICL1. Installation of phosphomimetic residues at GRK1 autophosphorylation sites in its AST improved catalytic efficiency and the yield of its crosslinked complex with Rho, likely by complementing the basic residues in ICL1 (52).

## The GRK1 membrane sensor

Membrane interactions play a key role in regulating the activity of GRKs toward GPCRs (70). While pursuing the structure of the Rho–GRK1 complex, a patch of positively charged residues immediately following the GRK1 αN helix was shown to promote GRK activity in a PIP_2_-dependent manner (52). The analogous region of GRK5 is well known to be important for PIP_2_ interactions (49, 50). Thus, it seems that most if not all GRKs have two distinct membrane sensors (N and C terminal). The C terminal membrane sensors are highly variable and relatively nonspecific, whereas the N terminal membrane sensors, when present, involve highly basic sequences that engage lipids with negatively charged head groups in a region close to the TM core of the receptor (Fig. 4*C*).

## Molecular origins of GRK/arrestin bias?

A receptor that is intrinsically biased or bound to an agonist biased toward GRK/arrestin might be expected to adopt a conformation that would favor both GRK and arrestin binding. Although we make this simplistic assumption in our analysis, we acknowledge that such may not always be the case. In fact, Rho in complex with either transducin or arrestin adopts a nearly identical conformation, but its conformation seems different when in complex with GRK1 (18, 52, 71). Note however that Rho is not subjected to ligand bias because it has only one known ligand. Conceptually, a receptor conformation that favors GRK binding also does not need to favor arrestin binding when the arrestin engages solely through a tail interaction.

**Figure 6:**
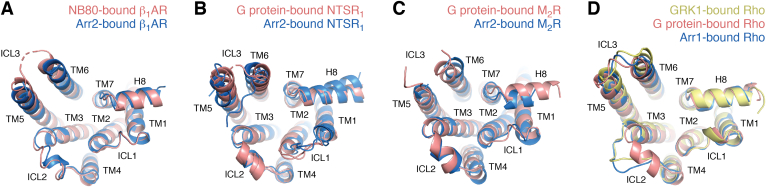
**Comparison of GPCRs bound to G proteins, GRK1, and arrestins show subtle differences but somewhat more condensed cytoplasmic clefts.***A*, overlay of G protein mimic nanobody (NB80) (PDB entry 6IBL) (36), Gs (PDB entry 7JJO) (73), and Arr2 (PDB entry 6TKO) (36) bound to the β_1_AR. *B*, overlay of G_i_ (PDB entry 6OSA and 6OS9) (78) and Arr2 (PDB entries 6PWC and 6UP7) (33, 34) bound to NTSR_1_. *C*, overlay of G_o_ (PDB entry 6OIK) (79) and Arr2 (PDB entry 6U1N) (35) bound to the Μ_2_R. *D*, overlay of GRK1 (PDB entry 7TMA) (52), G_t_ (PDB entry 6OYA) (71), and Arr1 (PDB entry 5W0P) (18) bound to Rho. β_1_AR, β1 adrenergic receptor; Arr1, arrestin-1; Arr2, arrestin-2; GPCR, G protein-coupled receptor; GRK, G protein-coupled receptor kinase; H8, helix 8; ICL, intracelluar loop; NTSR_1_, neurotensin receptor 1; Rho, rhodopsin; TM, transmembrane.

With these caveats in mind, we compared the conformation of specific GPCRs bound to either arrestins or GRKs with their conformation when bound to heterotrimeric G proteins to look for clues about what a GRK and/or arrestin-biased conformation might look like (Fig. 6). There are no large conformational differences in the cytoplasmic clefts of the receptors in these complexes, but a subtle theme does emerge. As noted in the β_1_AR–Arr2 and Rho–GRK1 structure determinations (36, 52), and also in a new structure of a GPCR intrinsically biased toward GRKs/arrestins (72), the cytoplasmic cleft is somewhat smaller relative to those of GPCRs in complex with G proteins, chiefly due to inward rotations of the cytoplasmic ends of TM5 and TM6 but also in some cases inward movement of the C-terminus of TM7 and/or H8. If a one GPCR ligand can manage to stabilize a more condensed cytoplasmic cleft than another, then it may therefore exert some bias for GRK/arrestin signaling. A caveat with this hypothesis is that the receptor elements involved in compression of the cytoplasmic pocket are known to be flexible. For example, compare the cytoplasmic ICL3 of the β_1_AR while in complex with the G protein mimic Nb80 and a heterotrimeric G protein (Fig. 6*A*) (36, 73). Thus, these regions can conform to whatever protein happens to be bound. The conformation of a receptor bound to a biased agonist would thus have to restrict inherent flexibility to enforce a “condensed cleft”. Changes in dynamics that control how often receptors sample a state with a more condensed cytoplasmic cleft in response to the binding of a biased ligand also very likely play a role and in fact, this is consistent with the high degree of heterogeneity observed so far in arrestin and GRK complexes with GPCRs. Despite these various hints, molecular basis of ligand bias however remains obscure, and we should brace for the possibility that bias for the GRK–arrestin pathway will ultimately result from the sum of many subtle conformational changes (74) along with dynamics that may turn out to be both receptor and phosphorylation pattern specific.

## Concluding thoughts

Structural analysis of GPCRs in complex with heterotrimeric G proteins has become relatively routine these days, thanks to useful tools that rigidify the nucleotide-free G proteins, but complexes with arrestin and GRK remain challenging because of their high intrinsic dynamics, conformational heterogeneity, and the multiple modes by which they can interact with activated receptors. Both GRKs and arrestins can exist in either core or tail-bound configurations, the latter of which cannot be studied at high resolution, at least yet, by cryo-EM. GPCR–GRK complexes are particularly challenging because of their low affinity relative to arrestin, a property not uncommon for protein kinases and their physiological substrates. Indeed, success in the Rho–GRK1 structure determination was likely achieved by manipulations that artificially enhanced affinity (as assessed by improvements in catalytic efficiency). Low affinity interactions often require the use of chemical crosslinking, protein fusions, or helper Fabs to enhance local concentration and facilitate biophysical studies, but of course, these approaches generate their own interpretational problems that must be considered. Although confounding with respect to understanding bias, the conformational heterogeneity observed in cryo-EM arrestin and GRK complexes is likely functionally relevant because it likely allows them to adapt to many different receptor and membrane contexts. Even if stabilizing a more condensed cytoplasmic cleft of an active GPCR turns out to be a general mechanism for achieving bias against heterotrimeric G proteins, the mechanisms by which a receptor intrinsically or a ligand allosterically promotes this state may likewise be as diverse as receptors themselves (*e.g.*, see discussion in (12)). Ligand-dependent control of receptor dynamics, such as those that control how often receptors sample such an GRK/arrestin-friendly conformation, are also anticipated to play a role in dictating pathway preference. Finally, because different phosphorylation patterns (barcodes) in polypeptides bound to arrestin have been shown to trigger different allosteric changes in remote regions of arrestin, which are in turn linked to diverse downstream signaling interactions (38, 75), we urgently need structures of nonvisual arrestins in complex with GPCRs with their native C-termini intact and modified by well-defined phosphosites. Only then, may we be able to fully understand the molecular consequences of barcoding.

## Conflict of interest

The authors declare that they have no conflicts of interest with the contents of this article.
